# Isolation and analysis of high quality nuclear DNA with reduced organellar DNA for plant genome sequencing and resequencing

**DOI:** 10.1186/1472-6750-11-54

**Published:** 2011-05-20

**Authors:** Kerry A Lutz, Wenqin Wang, Anna Zdepski, Todd P Michael

**Affiliations:** 1 Rutgers, The State University of New Jersey, Department of Plant Biology and Pathology, The Waksman Institute of Microbiology, Piscataway, NJ 08854, USA; 2 Current Address: Farmingdale State College, Ward Hall, Room 307, 2350 Broadhollow Road, Farmingdale, NY 11735, USA; 3 Monsanto Company, 800 North Lindbergh Blvd., Creve Coeur, Missouri 63167, USA

**Keywords:** chloroplast DNA, high throughput sequencing, mitochondrial DNA, nuclear DNA isolation, plant genome sequencing, quantitative real-time PCR (qPCR)

## Abstract

**Background:**

High throughput sequencing (HTS) technologies have revolutionized the field of genomics by drastically reducing the cost of sequencing, making it feasible for individual labs to sequence or resequence plant genomes. Obtaining high quality, high molecular weight DNA from plants poses significant challenges due to the high copy number of chloroplast and mitochondrial DNA, as well as high levels of phenolic compounds and polysaccharides. Multiple methods have been used to isolate DNA from plants; the CTAB method is commonly used to isolate total cellular DNA from plants that contain nuclear DNA, as well as chloroplast and mitochondrial DNA. Alternatively, DNA can be isolated from nuclei to minimize chloroplast and mitochondrial DNA contamination.

**Results:**

We describe optimized protocols for isolation of nuclear DNA from eight different plant species encompassing both monocot and eudicot species. These protocols use nuclei isolation to minimize chloroplast and mitochondrial DNA contamination. We also developed a protocol to determine the number of chloroplast and mitochondrial DNA copies relative to the nuclear DNA using quantitative real time PCR (qPCR). We compared DNA isolated from nuclei to total cellular DNA isolated with the CTAB method. As expected, DNA isolated from nuclei consistently yielded nuclear DNA with fewer chloroplast and mitochondrial DNA copies, as compared to the total cellular DNA prepared with the CTAB method. This protocol will allow for analysis of the quality and quantity of nuclear DNA before starting a plant whole genome sequencing or resequencing experiment.

**Conclusions:**

Extracting high quality, high molecular weight nuclear DNA in plants has the potential to be a bottleneck in the era of whole genome sequencing and resequencing. The methods that are described here provide a framework for researchers to extract and quantify nuclear DNA in multiple types of plants.

## Background

In many plant species, extracting large amounts of high quality, high molecular weight DNA can be a challenge due to high amounts of phenolic compounds, high levels of DNases and the presence of large amounts of organellar DNA (reviewed in [1]). Plant cells have three genomes, nuclear, plastid and mitochondrial. The number of organelles and genome copies per organelle depends on the species, cell type and age of the tissue. In *Arabidopsis thaliana*, a diploid plant species, each cell has two copies of each nuclear genome (gDNA). As the plant ages the number of genomes per cell can increase; the penultimate stage of rosette leaves has an average of 13 genome copies per cell [2]. The number of mitochondria and plastids can vary in different cell types. In an *Arabidopsis *root cell there are ~400 mitochondria [3], whereas maize anther cells have a 20 to 40-fold increase in the number of mitochondria [4]. In *Nicotiana sylvestris *there are more mitochondria in gametophytic cells than in other cell types [5]. Leaf cells of tobacco and pea leaves typically have ~100 chloroplasts and up to 10,000 plastid DNA (ptDNA) copies [6]. The number of ptDNA copies is also dependent on cell type; there is five times as much ptDNA in chloroplast-containing spinach cells than in amyloplast-containing cells [7]. In *Arabidopsis *and sugar beet the ptDNA copy number remains at ~1700 ptDNA copies per nuclear genome copy. The ratio of ptDNA to gDNA remains constant even as the ploidy level of the cell changes [2,8].

High throughput sequencing (HTS) is a relatively new application that has drastically reduced the cost of large scale sequencing and allowed scientists to generate a large amount of sequence in a short period of time. Researchers are leveraging HTS to sequence previously unsequenced genomes and resequence completed genomes. HTS was used to resequence the *Arabidopsis thaliana *ecotypes Columbia (Col), Bur-0 and Tsu-1 and identified over 800,000 single nucleotide polymorphisms, almost 80,000 1-3 bp indels and a region of 3.4 Mb that varied greatly, was missing or duplicated when compared to the to Col reference sequence [9]. A combination of Sanger sequencing and HTS was used to sequence grape [10] and a combination of Sanger, HTS and BAC sequencing was used for cucumber genome sequencing [11]. The gDNA of barley was sequenced with HTS, although only ~10% of the barley genome was sequenced [12].

HTS requires high quality, high molecular weight DNA because the first step in library preparation is to shear the DNA into small fragments by nebulization, sonication or enzymatic methods. Each method introduces sequence specific biases, which is exacerbated by degraded DNA and could result in loss of regions of the genome. In addition, cpDNA and mtDNA contamination in plants will directly impact the amount of nuclear genome sequence. For example, Illumina short read sequencing of several *Arabidopsis *ecotypes after DNA isolation with the DNeasy Plant Maxi Kit (Qiagen) resulted in 17.7% of the aligned reads being cpDNA or mtDNA [9]. Total cellular DNA isolated from barley by the CTAB protocol resulted in significant amounts of cpDNA and mtDNA in the DNA sample. This resulted in 60-fold coverage of the barley chloroplast genome and only ~10% of the barley nuclear genome being sequenced [12]. In general, plant genome projects aim for below 10% organellar contamination when plant material permits to maximize nuclear genome per sequencing dollar spent.

We describe optimized protocols for isolation of high quality, high molecular weight nuclear DNA from several species of plants with reduced levels of cpDNA and mtDNA contamination. We also developed a method that uses quantitative real time PCR (qPCR) to determine the level of cpDNA and/or mtDNA contamination in plant DNA preps. The DNA isolation protocols described here coupled with our analysis method will provide a framework for improving the nuclear DNA quality and quantity used for plant whole genome sequencing and resequencing.

## Methods

### Plant growth conditions

*Zea mays *(B73) and *Sorghum bicolor *(BTx623) plants were grown under standard greenhouse conditions. Young leaves from a two-month-old *Z. mays *plant and from a one-month-old *S. bicolor *plant were collected and flash frozen in liquid nitrogen. *Lemna gibba *(duckweed, JSP), *Spirodela polyrhiza *(Greater Duckweed, #7498), *Genlisea aurea *(corkscrew plant, CAP) and *Brachypodium distachyon *[13] (Bd21) plants were grown in a growth chamber under long day conditions (16 hours light at 22°C: 8 hours dark at 18°C). *L. gibba *and *S. polyrhiza *were grown in Schenk and Hildebrandt (Sigma, St. Louis, MO) media supplemented with 10 g/l sucrose (pH 5.8). *G. aurea *was grown in humus-rich wet soil mixed with sand and *B. distachyon *seed were stratified at 4°C for one week and the plants were grown in soil. Tissue from two-week-old *L. gibba *and *S. polyrhiza *plants, leaves from one-year-old *G. aurea *plants and young leaves from one-month-old *B. distachyon *plants were collected. Young leaf tissue was collected from *Vaccinium macrocarpon *(cranberry, CNJ03-228) grown in the greenhouse under natural light and temperature in late spring in Hammonton, NJ. *Arabidopsis thaliana *(*Col*) seed was sterilized, plated onto 1/2 MS (pH5.8) media, stratified at 4°C for 4 days and transferred to a constant light growth chamber with thermocycles (12 hours 22°C: 12 hours 12°C). Two weeks after seed plating, complete seedlings were collected. All tissue and leaf samples were flash frozen in liquid nitrogen.

### Total cellular DNA isolation with CTAB

0.25 g of plant tissue was flash frozen in liquid nitrogen and ground with a Retsch grinder. DNA was isolated using CTAB buffer as described in Murray *et al. *[14]. The CTAB isolated DNA was treated with 50 μg RNase for 30 minutes at 65°C and 120 μg Proteinase K at 45°C for one hour. The sample was extracted with chloroform:isoamyl alcohol (24:1) one time and precipitated with isopropanol.

### Nuclei isolation

Protocol A is used for plant tissue that contains less secondary metabolites or limited amount of plant material, such as *A. thaliana *and *G.aurea*. Nuclei were isolated using a sucrose gradient protocol modified from Gendrel et al. [15] as described in Table 1. Protocol A used 0.25 g of plant tissue that was flash frozen in liquid nitrogen and ground with a Retsch mixer mill (MM301; Haan, Germany). The ground tissue was resuspended in 30 ml ice-cold Extraction Buffer 1 (0.4 M Sucrose, 10 mM Tris, pH 8, 10 mM MgCl_2 _, 5 mM β-mercaptoethanol) and filtered through Miracloth. The sample was centrifuged for 20 minutes at 4000 rpm (1940*g*) at 4°C. The pellet was resuspend in 1 ml of chilled Extraction Buffer 2 (0.25 M Sucrose, 10 mM Tris, pH 8, 10 mM MgCl_2 _, 1% Triton X-100, 5 mM β-mercaptoethanol) and spun at 12,000*g *for 10 minutes at 4°C. The pellet was resuspend in 300 μl of chilled Extraction Buffer 3 (1.7 M Sucrose, 10 mM Tris, pH 8, 2 mM MgCl_2 _, 0.15% Triton X-100, 5 mM β-mercaptoethanol) and overlaid on top of 300 μl of chilled Extraction Buffer 3 and spun for 1 hour at 14,000*g *at 4°C. The nuclei pellet was resuspended in TE buffer (10 mM Tris-HCl, pH 8, 1 mM EDTA, pH 8). The DNA was treated with RNase and Proteinase K and the DNA was precipitated with isopropanol.

**Table 1:** Overview of nuclei isolation protocols A, B and C

	Protocol A	Protocol B	Protocol C
**Plant criteria**	•Low amount of plant material available •Low levels of secondary metabolites	•DNase-rich plants •Large amounts of plant tissue available	•High levels of secondary metabolites
**Successful DNA isolation **	•*A. thaliana* •*B. distachyon* •*G. aurea* •*S. bicolor* •L. gibba* •S. polyrhiza* •*Z. mays*	•*B. distachyon* •*L. gibba* •*S. bicolor* •*S. polyrhiza* •*Z. mays*	•V. *macrocarpon*
**Protocol Modifications**	•Used only 0.25 g tissue •Only performed DNA isolation steps (6-13) •Removed protease inhibitors from all buffers •Resuspended nuclei pellet in TE (10 mM:1 mM)	•Omitted TE slurry and diethyl ether steps •Increased volume of SEB to 200 ml •Added Triton X-100 drop by drop to minimize disruption of the nuclear membrane •Isolated DNA by isopropanol precipitation	•Omitted TE slurry and diethyl ether steps •Added EGTA and L-Lysine-HCl to MEB Buffer •Incubated sample on ice 8 minutes after addition of Triton X-100 •Centrifuged filtrate one time •Resuspended nuclei pellet in 1 ml of MPDB per gram of starting tissue
**Protocol modified from**	Gendrel *et. al. *[15]	Peterson *et. al. *[16] (Option Y)	Peterson *et. al. *[16] (Option X); Peterson *et. al. *[17]

* For *L. gibba *and *S. polyrhiza *the volume of buffer A had to be doubled for isolation on non-degraded DNA.

Protocol B was used for DNase-rich plant material, such as *L. gibba, S. polyrhiza*, and plant species where large amounts of starting material are available, such as *B. distachyon, Z. mays *and *S. bicolor*. This protocol was modified from [16] Option Y as described in Table 1. Protocol B used 10 g of ground tissue that was transferred into 200 ml fresh SEB extraction buffer (2.0% w/v polyvinylpyrrolidone (MW 40,000), 10% v/v TKE (Tris, KCl, EDTA: 0.1 M Tris base, 1.0 M KCl, 0.1 M EDTA, pH 9.4-9.5), 500 mM sucrose, 4 mM spermidine trihydrochloride, 1 mM spermine tetrahydrochloride, 0.1% w/v ascorbic acid, 0.13% w/v sodium diethyldithiocarbamate, 2.5% v/v β-mercaptoethanol). The mixture was placed on ice for 30 minutes and the homogenate was filtered through 2 layers of cheesecloth. Triton X-100 was added to a final concentration of 0.5%, the sample was placed on ice for 10 minutes, and then centrifuged at 650*g *for 15 minutes at 4°C. The nuclei pellet was resuspended in 100 ml SEB extraction buffer and centrifuged at 650*g *for 15 minutes at 4°C. The nuclei pellet was resuspended in TE and purified by digestion with RNase and Proteinase K; the DNA was precipitated with isopropanol.

Protocol C was used for plants that were rich in secondary metabolites such as *Vaccinium macrocarpon*. This protocol was modified from Option X from Peterson [16] and [17] as described in Table 1. Protocol C used 10 g ground tissue that was transferred into 200 ml fresh MEB extraction buffer (1 M 2-methyl-2,4-pentanediol (MPD), 10 mM PIPES-KOH, 10 mM MgCl_2 _, 2% PVP-10, 10 mM sodium metabisulfite, 5 mM β-mercaptoethanol, 0.5% sodium diethyldithiocarbamate, 6 mM EGTA, 200 mM L-lysine-HCl, pH 5.0). The homogenate was filtered through 2 layers of cheesecloth. Triton X-100 was added to a final concentration of 0.5%, placed on ice for 8 minutes and centrifuged at 800*g *for 20 min at 4°C. The pellet was resuspended in 100 ml of MPDB (0.5 M 2-methyl-2,4-pentanediol, 10 mM PIPES-KOH, 10 mM MgCl_2_.6H_2_O, 0.5% Triton X-100, 10 mM sodium metabisulfite, 5 mM β-mercaptoethanol, pH 7.0) and layered on top of a 37.5% Percoll bed (20 ml of 37.5% Percoll (7.5 ml percoll + 12.5 ml MPDB) in a 50 ml-centrifuge tube). The gradient was centrifuged in a swinging bucket rotor at 650*g *for 1 hour. The pellet was resuspended in 10 ml MPDB buffer, centrifuged at 300*g *for 10 minutes, and then centrifuged at 650*g *for an additional 10 min. The nuclei pellet was resuspended in TE and purified by digestion with RNase and Proteinase K; the DNA was precipitated with isopropanol.

### Quantitative Real Time PCR analysis

Quantitative real time PCR (qPCR) was carried out as described [18] using a myIQ single color quantitative Real Time PCR machine (BioRad). The thermocycling conditions were: denaturation at 95°C for 2 minutes and fourty cycles of 95°C for 15 seconds and 60°C for 20 seconds. Samples were assayed in triplicate (20 μl reactions) and the average C_t _value was calculated. The efficiency of the PCR reaction was determined using standard curves with a serial dilution of the template under investigation. The efficiency (E) of the reaction for each primer set was calculated for each DNA sample using the formula E = (10^-1/slope^) -1. One genomic gene, one chloroplast gene and one mitochondrial gene were amplified per DNA sample. Primer sets were designed for several nuclear, chloroplast and mitochondrial genes and preliminary experiments were performed to determine which primer pair yielded the best results (R^2 ^values > 0.9). For species where no mtDNA sequence was available no analysis was performed (*Brachypodium distachyon, Lemna gibba *and *Spirodela polyrhiza)*. In *G. aurea*, the efficiencies were calculated only for the CTAB DNA preparation due to limited plant material and low levels of DNA obtained from nuclei. The number of gene copies in a sample was determined by comparing the C_t _values from a DNA dilution curve with nuclei or CTAB isolated DNA. The following formula was used to calculate the number of chloroplasts relative to the number of genomic copies (gE^(gCt)^)/(cpE^(cpCt)^) or (gE^(gCt)^)/(mtE^(mtCt)^) for the number of mitochondria. To determine the percentage of cpDNA and mtDNA in each sample the following formula was used: ([{#cpDNA copies in nDNA}/ploidy number*cp genome size]/1C genome size)*100. Primer sequences were designed with the PRIMER EXPRESS V2.0 software (Applied Biosystems) and are shown in Additional file 1.

### Sequencing *G. aurea* using HTS

*G. aurea *DNA was prepared as described above and two standard SOLiD fragment libraries were constructed from either CTAB or nuclear DNA according to the manufacturer's protocol. Resulting libraries were amplified on beads by emulsion PCR and sequenced on the SOLiD v1 system (Life Technologies, Carlsbad, CA). Resulting colorspace reads were filtered for quality [19] and mapped to the genes used to make the qPCR primers through the corona lite mapping pipeline allowing 2 mismatches and 10 matches for any given read. Amount of gDNA, cpDNA and mtDNA was estimated based on the average coverage across each of the genes.

## Results

### DNA isolation protocols

**Figure 1:**
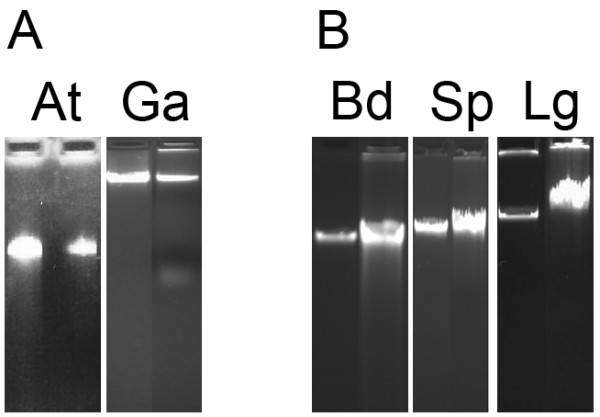
**Agarose gel analysis of high quality genomic DNA isolated from plant nuclei**. For each gel one microliter of genomic DNA was loaded onto a 0.8% agarose gel; the left panel is uncut lambda DNA and the right panel is the genomic DNA sample. A, Genomic DNA isolated using Protocol A: 50 ng lambda DNA, *Arabidopsis thaliana *(At) and 100 ng lambda DNA, *Genlisea aurea *(Ga). B, Genomic DNA isolated using Protocol B: 60 ng lambda DNA, *Brachypodium distachyon *(Bd), 60 ng lambda DNA, *Spirodela polyrhiza *(Sp), 125 ng lambda, *Lemna gibba *(Lg).

DNA was isolated from five monocot (*Brachypodium distachyon, Lemna gibba, Spirodela polyrhiza, Sorghum bicolor *and *Zea mays*) and three eudicot (*Arabidopsis thaliana, Genlisea aurea, Vaccinium macrocarpon*) plant species that sampled one woody (*Vaccinium macrocarpon*) and seven herbaceous (*Arabidopsis thaliana, Brachypodium distachyon, Genlisea aurea Lemna gibba, Spirodela polyrhiza, Sorghum bicolor *and *Zea mays*) plants. These species were chosen because they were either current genome projects or posed challenges to obtain high quality, high molecular weight DNA or nuclear DNA with low cp/mt contamination. Total cellular DNA was isolated from all species with the CTAB protocol. We also used several protocols (Protocol A, Protocol B and Protocol C) for isolation of nuclear DNA. Genomic DNA was isolated from all eight species with Protocol A. Protocol A yielded high quality DNA from *A. thaliana, B. distachyon *and *G. aurea *although the yield of DNA obtained from *G. aurea *was low due to the presence of polysaccharides in the sample [20]. The yield for *G. aurea *was ~0.8 micrograms per gram of starting material, 10 fold less that the yield obtained for *A. thaliana *(~10 micrograms per gram of tissue). The presence of polysaccharides in the *G. aurea *sample did not inhibit enzymatic reactions as DNA isolated by Protocol A was successfully sequenced using the SOLiD sequencer. To obtain non-degraded DNA from *L. gibba *and *S. polyrhiza *with Protocol A, two species with high DNase content, it was necessary to increase the volume of Buffer A from 30 ml to 60 ml. Protocol A did not yield high molecular weight DNA for *V. macrocarpon*. Protocol A is best for DNA isolation from plant tissue that contain less secondary metabolites or where limited amount of plant material is available. Figure 1A shows an agarose gel of genomic DNA isolated from *A. thaliana *and *G. aurea *with Protocol A.

Protocol B was attempted in species that gave difficulties in DNA isolation with Protocol A and also in species where large amounts of tissue were available, as the protocol requires 10 g of starting material. Protocol B yielded high quality DNA in all species tested (*L. gibba, S. polyrhiza, B. distachyon, Z. mays *and *S. bicolor*). Importantly, Protocol B yielded high quality DNA from *L. gibba *and *S. polyrrhiza*, two species that have high levels of DNase. Protocol B resulted in 20-40 micrograms of DNA per 10 grams of starting material. Figure 1B shows an agarose gel of genomic DNA isolated from *B. distachyon, L. gibba *and *S. polyrhiza *with Protocol B.

Protocol C was only attempted for DNA isolation from *V. macrocarpon*, a species that is rich in secondary metabolites. We obtained highly pure genomic DNA even though there were high levels of phenolics in the original tissue sample.

### Calculation of chloroplast, mitochondrial and nuclear DNA by qPCR

DNA samples isolated using one of the nuclei protocols (A, B or C) were compared to total cellular DNA isolated by CTAB (gDNA) to determine the level of cpDNA and mtDNA. Quantitative real time PCR (qPCR) was used to test the number of copies of cpDNA and mtDNA relative to the nuclear DNA (nDNA). qPCR analysis was performed using primers for a single copy nuclear gene, a single copy chloroplast gene and a mitochondrial gene (Additional file 1). The *GIGANTEA *(*Gi*) gene was used as a single copy reference in *A. thaliana *[21] and *B. distachyon *as only one copy of the gene is present. *Gi *was also used as a reference for *S. bicolor *and *Z. mays*, which both have two copies [22,23]. The *Gi *sequences for *S. polyrhiza *and *G. aurea *were obtained by PCR amplification with degenerate primers described in [24]. The single copy gene Dihydroflavonol-4-Reductase 2 (DFR2) was used for *V. macrocarpon*. A single copy chloroplast gene and mitochondrial gene was identified for each plant species. All chloroplast genes used were located outside of the inverted repeat region. The chloroplast genes used were: *rps18 *for *A. thaliana, rps16 *for *G. aurea, psbA *for *B. distachyon, S. bicolor *and *Z. mays, rbcL for S. polyrhiza and V. macrocarpon *and *matK *for *L. gibba*. The *cox1 *mitochondrial gene was used for *A. thaliana, G. aurea, S. bicolor *and *Z. mays *and *matR *was used for *V. macrocarpon*. Mitochondrial contamination was not tested in *B. distachyon, S. polyrhiza *and *L. gibba *due to the lack of a mitochondrial genome sequence.

gDNA and nDNA isolated from the different plant species was quantified with the Nanodrop 1000 (Nanodrop Technologies, Wilmington, DE) and a DNA dilution series (25 ng, 5 ng, 1 ng, 0.2 ng, 0.04 ng) was prepared for each sample. Each DNA dilution was performed in triplicate for each primer pair. The average C_t _value for every primer pair and DNA dilution were obtained and plotted against the log of the DNA concentration to obtain the slope of the graph needed to calculate the efficiency of the qPCR reaction. The results of the qPCR are listed in supplementary tables for *A. thaliana *(Additional file 2), *S. bicolor *(Additional file 3), *Z. mays *(Additional file 4), *V. macrocarpon *(Additional file 5), *L. gibba *(Additional file 6), *S. polyrhiza *(Additional file 7), *G. aurea *(Additional file 8) and *B. distachyon *(Additional file 9).

The number of cpDNA and mtDNA copies found in the gDNA and nDNA samples are shown in Table 2. Comparison of DNA obtained from nDNA (Protocols A, B, or C) with gDNA (isolated with CTAB) showed fewer cpDNA copies in the nDNA sample in all species tested. The reduction in cpDNA copies was relatively modest in some species; *B. distachyon*: 126 cpDNA copies in the CTAB sample and 107 copies in the nDNA samples (Table 2). Other species had a significant reduction in cpDNA contamination: *G. aurea *cpDNA copies reduced from 5467 in the CTAB sample to only 220 in the nDNA sample and *S. polyrhiza *showed a reduction from 778 cpDNA copies to only 72 in the nDNA sample (Table 2). The exact copy number of each sample is not necessarily meaningful as the exact values may change from experiment to experiment. Therefore, the percentage of cpDNA (or mtDNA) present in the DNA sample was calculated. This value determines the percentage of DNA in the preparation of cpDNA or mtDNA. The percentage of cpDNA present in the *L. gibba *DNA sample reduced to 1.3% for the nuclei DNA sample compared to 5.5% of the CTAB DNA sample (Table 2). A significant reduction of cpDNA copies was not seen for the *B. distachyon *samples, but the CTAB samples did not have a large amount of cpDNA contamination in them to start with (126 cpDNA copies; 2.8%).

**Table 2:** Copy number of chloroplast (Cp) and mitochondrial (Mt) genomes in DNA isolated with the CTAB protocol (gDNA) or from isolated nuclei (nDNA)

Plant	Nuclear genome size (Mb)	DNA isolation protocol	Organelle	gDNA (count)	nDNA (count)	gDNA (%)	nDNA (%)
*Arabidopsis thaliana*	150	A	Cp	317	192	15	9.1
			Mt	3	6		
*Brachypodium distachyon*	300	B	Cp	126	107	2.8	2.4
			Mt	NA	NA	NA	NA
*Genlisea aurea*	65	A	Cp	5467	220		
			Mt	93	7		
*Lemna gibba*	447	B	Cp	298	73	5.5	1.3
			Mt	NA	NA	NA	NA
*Sorghum bicolor*	692	B	Cp	1043	578	5.3	2.9
			Mt	52	43		
*Spirodela polyrhiza*	120	B	Cp	778	72		
			Mt	NA	NA	NA	NA
*Vaccinium macrocarpon*	450	C	Cp	3568	695		
			Mt	785	35		
*Zea mays*	2300	B	Cp	278	153	0.4	0.2
			Mt	16	6		

DNA was isolated from *Arabidopsis thaliana, Brachypodium distachyon, Genlisea aurea, Lemna gibba, Sorghum bicolor, Spirodela polyrhiza, Vaccinium macrocarpon *and *Zea mays*. Chloroplast DNA contamination in DNA isolated from nuclei or with CTAB. % cpDNA is calculated by ([{# cpDNA copies/#copies of genomic gene}* cp genome size]/1C genome size)*100. Numbers assume a chloroplast and mitochondria genome size of 150 and 500 kb respectively. Data not available (NA).

The results of qPCR analysis of CTAB isolated and nuclei isolated DNA for mtDNA contamination is shown in Table 2. All samples (except *A. thaliana*) showed a reduction in the number of mtDNA copies in the nuclei isolated DNA samples. There were 93 mtDNA copies in the *G. aurea *CTAB sample (Table 2) and only 7 copies in DNA isolated from nuclei. In comparison, *A. thaliana *did not show a decrease in mtDNA contamination (6 mtDNA copies in nuclei sample compared to 3 mtDNA copies in CTAB sample). Since there are so few mtDNA copies in the CTAB sample this slight increase is not statistically significant.

### Nuclei prep impact of high throughput sequencing

**Figure 2:**
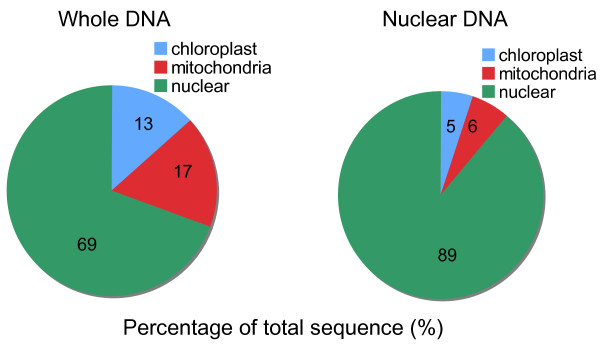
**Nuclear preparation of *Genlisea aurea *DNA reduces the percentage of chloroplast and mitochondrial genomes in short read sequencing experiments**. *Genlisea aurea *chloroplast genome (blue) is 150 kb, mitochondria genome (red) is 500 kb and nuclear genome (green) is 60,000 kb.

To determine the accuracy of our gDNA preparations and qPCR quantification, we sequenced CTAB and nuclear *G. aurea *DNA using HTS technology. *G. aurea*, better known as the corkscrew plant, and popularized by Charles Darwin in the last pages of his book on "insectivorous plants," [25] is reported to have the smallest angiosperm genome [26], which we have confirmed using flow cytometry (W. Wang and T.P. Michael, unpublished results). The corkscrew plant is indigenous to Africa and South America [27] and has modified leaves that burrow into the soil and twist in the form of a corkscrew where it traps all types of prey [28]. *G. aurea *is a carnivorous plant and may represent a minimal plant genome due to its mode of nutrient acquisition. Resulting short reads were mapped allowing two mismatches to the full-length genes used in the qPCR analysis. As expected the CTAB samples displayed a 100:50:1 (cp:mt:n) ratio consistent with the qPCR results. In contrast, the nuclei prep resulted in a 1:1:1 (cp:mt:n) ratio. Sequencing the gDNA sample resulted in 69% of the reads mapping to the nuclear genome, whereas 89% of the nDNA reads were from the nuclear genome (Figure 2).

## Discussion

We report here three protocols that yield high quality DNA from nuclei isolated from several monocot and eudicot plant species. The qPCR tools we designed to determine the purity of the genomic DNA sample are simplistic to use and will allow for identification of low quality DNA before entering the expensive and labor intensive short read sequencing protocol. Although we did not obtain DNA samples that are devoid of cpDNA and mtDNA, we did see a significant reduction in the number of cpDNA and mtDNA copies per genomic DNA copy. The amount of cpDNA copies was almost cut in half for sorghum (1043 copies for CTAB compared to 578 for nuclei isolation) and reduced by one-third for *A. thaliana *(317 copies for CTAB compared to 192 for nuclei isolation). The reduction in the number of mitochondrial genome copies was not as impressive (for example Sorghum mtDNA copies changed from 52 CTAB to 43 in the nuclei sample; Table 2), as there are fewer mtDNA copies in a cell relative to cpDNA copies. Isolation of nuclei will be beneficial in plant species with a significant decrease in cpDNA copies when compared to the CTAB samples. Such samples that would benefit from nuclei isolation are *A. thaliana, L. gibba, G. aurea, S. polyrhiza *and *V. macrocarpon*. Since maize and sorghum have relatively large nuclear genomes, the benefit of reducing the cpDNA number may not be as obvious. Both *Z. mays *and *S. bicolor *had a 50% drop in cpDNA copies when DNA was isolated with nuclei isolation yet *Z. mays *only saw a 0.2% and *S. bicolor *only had a 2.4% decrease in cpDNA copies (Table 2).

All three of the nuclei isolation protocols reduce the amount of cpDNA and mtDNA present in most plant species tested. Identification of which protocol to use is dependent on the amount of secondary metabolites as well as the amount of starting material. Protocol A uses Triton X-100 for preferential elimination of chloroplasts and mitochondria. We found Protocol A to be suitable for samples with low amounts of available starting material. Best results were obtained with Protocol A for *G. aurea *and *A. thaliana*. We also obtained high quality DNA for *L. gibba *and *S. polyrrhiza*, but the volume of Buffer A had to be doubled or the DNA samples were degraded. Protocol B is ideal for samples with high levels of DNases, as the extraction buffer contains EDTA, which efficiently inhibits the activity of DNase. Protocol B yielded high quality DNA from all samples tested, *L. gibba, S. polyrhiza, B. distachyon, Z. mays *and *S. bicolor*. This protocol is only suitable when large amounts of starting material are available. The final DNA yield for Protocol A is 2.5 micrograms (~10 micrograms per gram of starting material) whereas Protocol B yields 20-40 micrograms (~2-4 micrograms of DNA per gram of starting material). The extraction buffer in Protocol C is composed of the antioxidants β-mercaptoethanol, sodium diethyldithiocarbamate, sodium metabisulfite, and has a lower pH to limit the oxidation of polyphenols; it also has polyvinylpyrrolidone, which adsorbs polyphenols to prevent them from interacting with DNA [29]. Protocol C is best for species that have high levels of secondary metabolites, such as *V. macrocarpon *(cranberry)*, Mentha requienii *(mint)*, Ocimum basilicum *(basil) *and Fragaria vesca *(alpine strawberry). It may have been advantageous to test *G. aurea *with Protocol C, due to the presence of polysaccharides, but due to limited amounts of tissue this experiment was not performed. Recently, Carrier *et. al. *[30] describe a nuclei isolation protocol for woody plants with high polysaccharides. Their protocol is similar to our Protocol C, in that they use PVP and β-mercaptoethanol for prevention of polyphenol oxidation, it differs because they use a sucrose gradient for nuclei isolation and silica column for DNA isolation. Polysaccharides may be particularly problematic when present in DNA samples, as their presence can inhibit enzymatic activity. Presence of polysaccharides has been shown to inhibit Taq polymerase activity [31] and restriction enzyme activity [32]. The presence of polysaccharides in the DNA sample is characterized by formation of a highly viscous solution [33].

Using different tissue types or modifying the growth conditions can also achieve further reduction in organellar DNA copies. Lower levels of starch can be obtained in *Fragaria *(strawberry) by growing then under short day conditions and then shifting the plants to the dark. In *Solanum nigrum *cpDNA content can be reduced by growing cultures in the presence of DNA gyrase inhibitors [34]. For all of our experiments young leaf tissue was used. An alternate method to increase genomic DNA yield is to use older leaf tissue. In *A. thaliana*, examination of the nuclear and plastid DNA content of tissue isolated from plants of different developmental ages (2- day-old cotyledons to 37-day-old senescent rosette leaves) showed an increase in nuclear DNA copy numbers (up to 128 genome copies per nucleus in older leaves). Whereas, the plastome copy numbers did not show significant differences during development from young to old rosette leaves [2]. Several recent papers show a decrease in the ptDNA level as the plant ages [6,35,36], although these results are inconsistent. Therefore it is unclear whether ptDNA levels decrease throughout development of the plant. However, we observed lower amounts of high molecular weight DNA when isolated from older leaf tissue (data not shown) therefore use of younger plant tissues is recommended.

Reduction of the cpDNA in the DNA sample will directly result in increased coverage of the nuclear genome. A pure nDNA sample would reduce the amount of chloroplast and mitochondrial reads therefore increasing the coverage of the nuclear genome. By isolating *G. aurea *DNA from nuclei we obtained 20% more reads from nuclear DNA than from the sample isolated with CTAB. In species where nDNA isolation is not feasible, one can increase the number of HTS reads to increase the amount of nDNA sequenced. We were unable to eliminate all cpDNA and mtDNA from the nDNA samples. This can be an advantage becuase the cpDNA can be assembled to determine the quality of the HTS experiment. We were able to assemble the cpDNA and mtDNA sequences from the *G. aurea *gDNA and nDNA samples, although we needed 150x sequence of the nDNA to get the same coverage of organellar DNA. The DNA isolation protocols we describe here, coupled with the qPCR approach to check the quality of the gDNA will be beneficial to scientists performing short read sequencing experiments in plants. These techniques will improve the quality of the sequencing library as well as improve the coverage obtained of the genomic DNA. This will result in generation of more nuclear DNA reads from fewer runs that will yield higher genome coverage for bioinformatic analysis of the short read data.

## Conclusions

Using DNA prepared from isolated nuclei resulted in DNA samples with reduced numbers of cpDNA and mtDNA copies. This reduction was validated by using a new qPCR protocol to determine the level of organellar DNA contamination. This high quality DNA will be essential for improving the coverage obtained with short read sequencing therefore improving the bioinformatic analysis of the data.

## Authors' contributions

KL designed and performed DNA isolation and qPCR for *A. thaliana Z. mays S. bicolor, L. gibba, S. polyrhiza *and *B. distachyon *and drafted the manuscript. WW designed and performed DNA isolation and qPCR experiments for *Z. mays, S. bicolor, L. gibba, S. polyrhiza, G. aurea *and *B. distachyon*. AZ designed and performed DNA isolation protocol for *V. macrocarpon*. TM conceived of the study and performed bioinformatic analysis of HTS reads. All authors have read and approved the manuscript.

## Supplementary Material

Additional file 1 **qPCR primers**. A list of primers used for qPCR analysis. Click here for file

Additional file 2 **qPCR results of *Arabidopsis thaliana *nuclei and CTAB isolated DNA with nuclear (*Gi*), mitochondrial (*cox1*) and chloroplast (*rps18*) primers**. qPCR results for *Arabidopsis thaliana*. Table contains the diluted DNA concentrations, with the corresponding Ct values as well as the calculated for each primer pair qCR reaction and number of organelles per diploid genome. Click here for file

Additional file 3 **qPCR results of *Sorghum bicolor *nuclei and CTAB isolated DNA with nuclear (*Gi*), mitochondrial (*cox1*) and chloroplast (*psbA*) primers**. qPCR results for *Sorghum bicolor*. Table contains the diluted DNA concentrations, with the corresponding Ct values as well as the calculated efficiencies for each primer pair qPCR reaction and number of organelles per diploid genome. Click here for file

Additional file 4 **qPCR results of *Zea mays *nuclei and CTAB isolated DNA with nuclear (*Gi*), mitochondrial (*cox1*) and chloroplast (*psbA*) primers**. qPCR results for *Zea mays*. Table contains the diluted DNA concentrations, with the corresponding Ct values as well as the calculated efficiencies for each primer pair qPCR reaction and number of organelles per diploid genome. Click here for file

Additional file 5 **qPCR results of *Vaccinium macrocarpon *nuclei and CTAB isolated DNA with nuclear (*Dfr2*), mitochondrial (*matR*) and chloroplast (*rbcL*) primers**. qPCR results for *Vaccinium macrocarpon*. Table contains the diluted DNA concentrations, with the corresponding Ct values as well as the calculated efficiencies for each primer pair qPCR reaction and number of organelles per diploid genome. Click here for file

Additional file 6 **qPCR results of *Lemna gibba *nuclei and CTAB isolated DNA with nuclear (*Gi*) and chloroplast (*matK*) primers**. qPCR results for *Lemna gibba*. Table contains the diluted DNA concentrations, with the corresponding Ct values as well as the calculated efficiencies for each primer pair qPCR reaction and number of organelles per diploid genome. Click here for file

Additional file 7 **qPCR results of *Spirodela polyrhiza *nuclei and CTAB isolated DNA with nuclear (*Gi*) and chloroplast (*rbcL*) primers**. qPCR results for *Spirodela polyrhiza*. Table contains the diluted DNA concentrations, with the corresponding Ct values as well as the calculated efficiencies for each primer pair qPCR reaction and number of organelles per diploid genome. Click here for file

Additional file 8 **qPCR results of *Genlisea aurea *nuclei and CTAB isolated DNA with nuclear (*Gi*), mitochondrial (*cox1*) and chloroplast (*rps16*) primers**. qPCR results for *Genlisea aurea*. Table contains the diluted DNA concentrations, with the corresponding Ct values as well as the calculated efficiencies for each primer pair qPCR reaction and number of organelles per diploid genome. Click here for file

Additional file 9 **qPCR results of *Brachypodium distachyon *nuclei and CTAB isolated DNA with nuclear (*Gi*) and chloroplast (*psbA*) primers**. qPCR results for *Brachypodium distachyon*. Table contains the diluted DNA concentrations, with the corresponding Ct values as well as the calculated efficiencies for each primer pair qPCR reaction and number of organelles per diploid genome. Click here for file
