# Supplementary material for: Isolation and analysis of high quality nuclear DNA with reduced organellar DNA for plant genome sequencing and resequencing
Source: BMC Biotechnol. 2011 May 20;11:54. doi: 10.1186/1472-6750-11-54 (PMC3131251; doi:10.1186/1472-6750-11-54)
Supplement: Additional file 1 — qPCR primers. A list of primers used for qPCR analysis. [file 1472-6750-11-54-S1.DOC]

**Supplementary Table S1. qPCR primers.**

| **Organism** | **Gene Name** | **Primer Sequence (5'-3')** |
| --- | --- | --- |
| **Bd** | NucF Gi 357 | TTACGTGGAGTACTTTGGCCAGTT |
|  | NucR Gi 408 | TTCAGGGAATTGCTCGCTG |
|  | CpF psbA 432 | TGTTGCATATTCAGCTCCTGTTG |
|  | CpR psbA 483 | GTAAATCAAGAAAACAGCAGTCGC |
|  |  |  |
| **Zm** | NucF Gi 2617 | AACTTCCTTACCATGGATCGGA |
|  | NucR Gi 2667 | AAGAGTTTGTGAACCTCGGTAACC |
|  | CpR psbA 597 | CATGTGAAATGGATGCATAAGGAT |
|  | CpF psbA 547 | ATGATTGTATTCCAGGCAGAGCA |
|  | MtF cox1 718 | TTCTTCGGTCATCCAGAGGTGTATA |
|  | MtRcox1 768 | AATACCGAATCCAGGCAGAATG |
|  |  |  |
| **Sb** | NuF Gi 1820 | ATGGTTTGCTACCGCTAAGTTCA |
|  | NuR Gi 1870 | CAACAATTTCAGGTAAATCAACTGCT |
|  | CpF psbA 61 | ATAACTAGCACCGAAAACCGTCTT |
|  | CpR psbA 111 | CATCAAAACACCGAACCATCC |
|  | MtF cox1 1443 | AAAGAACAAAAGATGTGCGGAAAG |
|  | MtR cox1 1493 | GTTGGATTCTGCTCAACAGCC |
|  |  |  |
| **At** | NuF Gi 1203 | ACATGCTTTGATACAGCGGTGA |
|  | NuR Gi 1253 | TGGATTCATTTCAGTCCTTGAGG |
|  | CpF rps18 |  |
|  | CpR rps18 |  |
|  | MtF cox1 719 | TCTTCGGTCATCCAGAGGTGTA |
|  | MtR cox1 769 | TTCTCTCTAATGTCAATGCACGC |
|  |  |  |
| **Lg** | NuF Gi 3849 | TGACTCTGCATCTGTGTTCTTGC |
|  | NuR Gi 3900 | TTCTCTCTAATGTCAATGCACGC |
|  | CpF matK 1311 | TAGACTTTCCTGTGCTCGAACTTTG |
|  | CpR matK 1361 | CGTACTGTACTTTTATGTTTACGAGCCA |
|  |  |  |
| **Ga** | NuF Gi | CTGCTTCCACGGAAAGAAAC |
|  | NuR Gi | AACCGGTGGTAACAGCTGAC |
|  | CpF rps16 | CGTGCGTGAATCAACTGTCT |
|  | CpR rps16 | GGTAGCAACGGACCCTTTTT |
|  | MtF cox1 | TGGGCACATGCTTTTCAGTA |
|  | MtR cox1 | CAAGAATTTGATCGCCAGGT |
|  |  |  |
| **Vm** | NuF Dfr2 960 | AAATGTGTCGTTTCCGTCGAA |
|  | NuR Dfr2 1010 | TTGAACTGAAA CCCCATCCC |
|  | CpF rbcL 578 | TGATGAAAACGTGAACTCCCAA |
|  | CpR rbcL 628 | AAGAAACGGTCTCTCCAACGC |
|  | MtF matR 487 | TCAACGCTCCCCAAGACAAG |
|  | MtR matR 537 | TGCTCTTTACCCTCCCCACA |
|  |  |  |
| **Sp** | NuF Gi | ATGGATTGCCCGAGATGATGA |
|  | NuR Gi | AATTTGACGATGCGCTGGTTGAT |
|  | CpF | GCTGCCGAATCTTCTACTGG |
|  | CpR | CGATCAAGGCTGGTAAGTCC |
