# Supplementary material for: Isolation and analysis of high quality nuclear DNA with reduced organellar DNA for plant genome sequencing and resequencing
Source: BMC Biotechnol. 2011 May 20;11:54. doi: 10.1186/1472-6750-11-54 (PMC3131251; doi:10.1186/1472-6750-11-54)
Supplement: Additional file 5 — qPCR results of Vaccinium macrocarpon nuclei and CTAB isolated DNA with nuclear (Dfr2), mitochondrial (matR) and chloroplast (rbcL) primers. qPCR results for Vaccinium macrocarpon. Table contains the diluted DNA concentrations, with the corresponding Ct values as well as the calculated efficiencies for each primer pair qPCR reaction and number of organelles per diploid genome. [file 1472-6750-11-54-S5.doc]

**Supplementary Table S5. qPCR results of *Vaccinium macrocarpon* nuclei and CTAB isolated DNA with nuclear (*Dfr2*), mitochondrial (*matR*) and chloroplast (*rbcL*) primers.**

| **Avg C_T_ Value** | **log[DNA]** | **Sample Name** | **Eff.** | **# organelles /diploid gen.** |
| --- | --- | --- | --- | --- |
| 23.44 | 1.40 | Vm Nuclei 25 ng dfr2 | 2.02 |  |
| 25.72 | 0.70 | Vm Nuclei 5 ng dfr2 |  |  |
| 28.15 | 0.00 | Vm Nuclei 1 ng dfr2 |  |  |
| 30.43 | -0.70 | Vm Nuclei 0.2 ng dfr2 |  |  |
| 32.50 | -1.40 | Vm Nuclei 0.04 ng dfr2 |  |  |
|  |  |  |  |  |
| 18.14 | 1.40 | Vm Nuclei 25 ng matR | 2.04 | 35.39 |
| 20.48 | 0.70 | Vm Nuclei 5 ng matR |  |  |
| 22.72 | 0.00 | Vm Nuclei 1 ng matR |  |  |
| 25.06 | -0.70 | Vm Nuclei 0.2 ng matR |  |  |
| 27.12 | -1.40 | Vm Nuclei 0.04 ng matR |  |  |
|  |  |  |  |  |
| 14.36 | 1.40 | Vm Nuclei 25 ng rbcL | 2.00 | 695.39 |
| 16.46 | 0.70 | Vm Nuclei 5 ng rbcL |  |  |
| 19.44 | 0.00 | Vm Nuclei 1 ng rbcL |  |  |
| 21.53 | -0.70 | Vm Nuclei 0.2 ng rbcL |  |  |
| 23.40 | -1.40 | Vm Nuclei 0.04 ng rbcL |  |  |
|  |  |  |  |  |
| 27.78 | 1.40 | Vm CTAB 25 ng dfr2 | 2.21 |  |
| 30.14 | 0.70 | Vm CTAB 5 ng dfr2 |  |  |
| 32.55 | 0.00 | Vm CTAB 1 ng dfr2 |  |  |
| 34.83 | -0.70 | Vm CTAB 0.2 ng dfr2 |  |  |
| 35.59 | -1.40 | Vm CTAB 0.04 ng dfr2 |  |  |
|  |  |  |  |  |
| 21.57 | 1.40 | Vm CTAB 25 ng matR | 2.04 | 785.37 |
| 23.97 | 0.70 | Vm CTAB 5 ng matR |  |  |
| 26.33 | 0.00 | Vm CTAB 1 ng matR |  |  |
| 28.59 | -0.70 | Vm CTAB 0.2 ng matR |  |  |
| 30.56 | -1.40 | Vm CTAB 0.04 ng matR |  |  |
|  |  |  |  |  |
| 19.99 | 1.40 | Vm CTAB 25 ng rbcL | 2.00 | 3568.71 |
| 22.49 | 0.70 | Vm CTAB 5 ng rbcL |  |  |
| 25.43 | 0.00 | Vm CTAB 1 ng rbcL |  |  |
| 27.17 | -0.70 | Vm CTAB 0.2 ng rbcL |  |  |
| 29.27 | -1.40 | Vm CTAB 0.04 ng rbcL |  |  |
