# Supplementary material for: Isolation and analysis of high quality nuclear DNA with reduced organellar DNA for plant genome sequencing and resequencing
Source: BMC Biotechnol. 2011 May 20;11:54. doi: 10.1186/1472-6750-11-54 (PMC3131251; doi:10.1186/1472-6750-11-54)
Supplement: Additional file 9 — qPCR results of Brachypodium distachyon nuclei and CTAB isolated DNA with nuclear (Gi) and chloroplast (psbA) primers. qPCR results for Brachypodium distachyon. Table contains the diluted DNA concentrations, with the corresponding Ct values as well as the calculated efficiencies for each primer pair qPCR reaction and number of organelles per diploid genome. [file 1472-6750-11-54-S9.doc]

**Supplementary Table S9. qPCR results of *Brachypodium distachyon* nuclei and CTAB isolated DNA with nuclear (*Gi*) and chloroplast (*psbA*) primers.**

| **Avg C_T_ Value** | **log[DNA]** | **Sample Name** | **Eff.** | **# organelles /diploid gen.** |
| --- | --- | --- | --- | --- |
| 22.52 | 1.40 | Bd Nuclei 25 ng Gi | 2.06 |  |
| 24.99 | 0.70 | Bd Nuclei 5 ng Gi |  |  |
| 27.01 | 0.00 | Bd Nuclei 1 ng Gi |  |  |
| 29.59 | -0.70 | Bd Nuclei 0.2 ng Gi |  |  |
| 31.31 | -1.40 | Bd Nuclei 0.04 ng Gi |  |  |
|  |  |  |  |  |
| 17.01 | 1.40 | Bd Nuclei 25 ng psbA | 1.99 | 107.99 |
| 19.13 | 0.70 | Bd Nuclei 5 ng psbA |  |  |
| 21.87 | 0.00 | Bd Nuclei 1 ng psbA |  |  |
| 24.57 | -0.70 | Bd Nuclei 0.2 ng psbA |  |  |
| 26.02 | -1.40 | Bd Nuclei 0.04 ng psbA |  |  |
|  |  |  |  |  |
| 22.78 | 1.4 | Bd CTAB 25 ng Gi | 2.09 |  |
| 24.77 | 0.7 | Bd CTAB 5 ng Gi |  |  |
| 27.08 | 0 | Bd CTAB 1 ng Gi |  |  |
| 29.31 | -0.7 | Bd CTAB 0.2 ng Gi |  |  |
| 31.41 | -1.4 | Bd CTAB 0.04 ng Gi |  |  |
|  |  |  |  |  |
| 17.07 | 1.40 | Bd CTAB 25 ng psbA | 2.02 | 126.37 |
| 19.20 | 0.70 | Bd CTAB 5 ng psbA |  |  |
| 21.87 | 0.00 | Bd CTAB 1 ng psbA |  |  |
| 24.12 | -0.70 | Bd CTAB 0.2 ng psbA |  |  |
| 26.08 | -1.40 | Bd CTAB 0.04 ng psbA |  |  |
